# Keller/eGFR ratio as a simple and useful tool to make a first differentiation between renal aging and chronic nephropathy in large populations

**DOI:** 10.1007/s11255-025-04932-1

**Published:** 2025-12-03

**Authors:** Fabrizio Cristiano, Gustavo Aroca-Martinez, Carlos Guido Musso

**Affiliations:** 1 Department of Neuroscience, Imaging and Clinical Science, Gabriele d’Annunzio University of Chieti and Pescara, Chieti, Italy; 2 Nephrology and Dialysis Unit, Ortona Hospital, ASL 2 Lanciano Vasto, Chieti, Italy; 3 Facultad de Ciencias de la Salud, Universidad Simon Bolivar, Barranquilla, Colombia; 4 Research Department, Universidad Hospital Italiano, Buenos Aires, Argentina

**Keywords:** Renal ageing, Chronic kidney disease, eGFR, Keller formula, CKD overdiagnosis, Population screening

## Abstract

**Background:**

Chronic kidney disease (CKD) prevalence increases with age, but distinguishing physiological renal ageing from pathological CKD remains a major diagnostic challenge. Current CKD definitions based solely on estimated glomerular filtration rate (eGFR) often lead to overdiagnosis in elderly individuals with normal age-related decline. This study explores the use of the Keller/eGFR ratio as a simple and useful tool to differentiate normal renal ageing from true nephropathy in large populations.

**Methods:**

Keller formula (GFR = 130—age) was applied to model the expected physiological GFR decline across the lifespan. The ratio between Keller-derived GFR and measured eGFR (CKD-EPI) was analyzed to distinguish physiological ageing (Keller/eGFR ≤ 1) from pathological decline (Keller/eGFR > 1). The approach was compared with classical biochemical, urinary, and imaging markers and validated using data from the Abruzzo cohort of older adults.

**Results:**

Keller/eGFR ratio provided a clear separation between individuals with expected age-related GFR reduction and those with evidence of underlying CKD. In patients with Keller/eGFR ≤ 1, biochemical parameters (creatinine, urea, hemoglobin, calcium–phosphorus metabolism) and urinalysis remained within normal limits, and imaging findings were unremarkable. Conversely, Keller/eGFR > 1 was associated with typical CKD features, including anemia, mineral abnormalities, and structural renal alterations. Integrating this ratio with clinical and laboratory data significantly reduced CKD overdiagnosis in elderly subjects.

**Conclusions:**

The Keller/eGFR ratio represents a practical, low-cost, and easily applicable index for the first-line screening of kidney function in older adults. When combined with biochemical and imaging markers, it enhances diagnostic accuracy and helps avoid excessive medicalization related to CKD misclassification. Wider implementation in population studies could improve epidemiological stratification and resource allocation in nephrology care.

## Introduction

Chronic kidney disease (CKD) is a condition with a high epidemiological, clinical, and socio-health impact, with an estimated prevalence of approximately 10–12% in the general adult population. This prevalence increases exponentially with age, reaching values above 30–35% in individuals aged ≥ 65 years. This rise reflects, at least in part, the higher incidence of renal and cardiovascular comorbidities in the geriatric population, but it also results from the intrinsic difficulty in distinguishing normal renal aging from underlying CKD [1]. Such decline, which has been attributed to mechanisms including ageing-associated nephrosclerosis, reduced basal metabolic rate, and sarcopenia, should not necessarily be regarded as evidence of kidney disease but rather as a normal manifestation of renal ageing. Nevertheless, a subset of individuals exhibit what has been termed “successful renal ageing,” characterized by the preservation of glomerular filtration rate (GFR) without a clinically relevant decline [2]. The distinction between normal renal ageing and CKD in older individuals is, however, challenging in clinical practice, since both conditions may present with estimated GFR (eGFR) values below 60 ml/min/1.73 m^2^. Commonly used CKD diagnosis solely based on eGFR, not taking into account other parameters (creatininemia, hemoglobin, urinalysis, renal imaging, etc.), tends to overdiagnose CKD in the old population [3]. The clinical consequences of CKD overdiagnosis in older people should not be underestimated. Mislabeling a patient as affected by CKD may lead to inappropriate therapeutic interventions, including low-protein diets or the use of antiproteinuric/antifiltration agents (renin–angiotensin system inhibitors), with the risk of accelerating sarcopenia, inducing malnutrition, and precipitating fluid and electrolytes disorders, particularly hyperkalemia. Moreover, excessive medicalization of this population entails both direct and indirect costs for the healthcare system, as well as a potential deterioration in the quality of life of older adults [3, 4].

## Normal renal ageing

Normal renal ageing represents a universal, progressive, and multifactorial process, characterized by a gradual decline in GFR beginning in the fourth decade of life. Longitudinal studies have shown that GFR decreases by an average of approximately 0.75–1 ml/min/1.73 m^2^ per year after the age of 40, even in the absence of overt kidney disease [5]. This reduction should not be automatically interpreted as a sign of CKD, but rather as an expression of so-called “normal renal ageing.” The ageing mechanism underlying this decline involves both structural and functional alterations. From a histopathological perspective, renal ageing is characterized by ageing-associated nephrosclerosis, with thickening of the glomerular basement membranes, segmental hyalinosis, and a reduction in the number of functioning nephrons [6].In the glomerular compartment, renal ageing is characterized by basement membrane thickening, segmental glomerulosclerosis, and a progressive reduction in the number of functional nephrons. Within the tubulointerstitial compartment, age-related changes include interstitial fibrosis and peritubular capillary rarefaction, both of which critically contribute to the progressive decline in renal functional reserve [7]. With advancing age, there is also a progressive decline in skeletal muscle mass (sarcopenia) and basal metabolic rate (BMR). These changes alter creatinine generation and turnover, thereby limiting the reliability of serum creatinine as a biomarker and complicating accurate estimation of kidney function in older individuals [8]. A distinctive feature of age-related GFR decline is that it is not necessarily accompanied by the biochemical or clinical abnormalities typically observed in CKD. In patients with normal renal ageing, serum levels of creatinine, urea, hemoglobin, calcium, phosphate, and parathyroid hormone generally remain within the normal range, and both urinalysis and renal imaging are unremarkable. These findings help differentiate normal ageing of the kidney from CKD [9]. A minority of the population, described as exhibiting “successful renal ageing,” maintains a relatively preserved GFR even at very advanced ages, suggesting the presence of protective genetic and environmental factors that remain incompletely understood. However these determinants may have significant implications for the prevention and management of renal ageing [10].

## Chronic kidney disease in older adults

To KDIGO guidelines, CKD is defined by the presence, for at least three months, of an eGFR < 60 ml/min/1.73 m^2^ or by evidence of kidney damage. The latter includes persistent albuminuria, urinary sediment abnormalities, structural alterations on imaging, or disturbances in mineral and hemoglobin metabolism [11]. Although this definition is widely accepted and standardized, its application to the geriatric population raises important concerns, as GFR decline in older adults may reflect normal ageing rather than true CKD. Distinguishing features that help differentiate pathological CKD from normal renal ageing include the presence of significant proteinuria (particularly albuminuria > 30 mg/g), structural abnormalities on renal imaging (such as loss of cortico-medullary differentiation, multiple cysts, or advanced nephroangiosclerosis), disturbances of calcium–phosphate metabolism, and renal anemia—findings that are always absent in normal renal ageing [12]. Therefore, in older adults, the diagnosis of CKD cannot be based only on a reduced estimated GFR. A reliable assessment requires integration of biomarkers, urinalysis, renal imaging, and a comprehensive clinical evaluation, in order to minimize the risk of CKD overdiagnosis and avoid potentially inappropriate therapeutic intervention Additionally, a critical issue in the evaluation of kidney function in older adults is the accuracy of GFR estimating equations. Commonly employed creatinine-based formulas, such as MDRD and CKD-EPI, often overestimate GFR in sarcopenic individuals, thereby increasing the likelihood of CKD underdiagnosis. This limitation arises because reduced muscle mass in advanced age lowers creatinine generation, weakening the link between serum creatinine levels and true glomerular filtration capacity [13]. Alternative approaches have been proposed, most notably the use of cystatin C, a low–molecular weight protein produced at a constant rate by all nucleated cells and freely filtered at the glomerular level [14]. Several studies have shown that GFR estimates based on cystatin C—or on combined creatinine–cystatin C equations—provide greater accuracy in assessing kidney function in older individuals, mitigating the biases introduced by sarcopenia and dietary variability [15, 16]. However, cystatin C is not without limitations, as its levels may be affected by chronic inflammation, thyroid dysfunction, and obesity [17].

## Keller’s formula and the Keller/eGFR ratio

The formula proposed by Keller in the late 1980s provided a simple tool to describe the normal age-related decline in kidney function. According to this formula (GFR = 130 – age [years]), GFR is assumed to decrease by approximately 1 ml/min/1.73 m^2^ per year after the fourth decade of life [18]. Keller’s formula (GFR = 130—age) does not stem from a single peer-reviewed publication authored by Keller. Rather, it reflects an empirical model introduced in the 1980s in German and Swiss nephrology teaching as a practical representation of age-related GFR loss. Its rationale is consistent with classic longitudinal data showing a decline of ~ 0.75–1 ml/min/1.73 m^2^ per year after the fourth decade [18]. Although it is an empirical model not derived from large multicenter cohorts, Keller’s formula mathematically reflects the already documented GFR reduction rate associated with normal ageing, and consequently remains a valuable physiological reference, as it helps distinguish normal renal ageing from CKD. An alternative age-related GFR model is the Rutkowski formula, presented in the NKF-KDOQI report on kidney function assessment in the elderly. Derived from Polish cohorts, it describes a linear decline in GFR with ageing that closely parallels rates reported in classical longitudinal studies. Although less commonly used in clinical practice than equations developed by large international consortia, the Rutkowski model provides a useful comparative reference and supports the physiological interpretation of the age-related GFR decline adopted in this manuscript. Considering both Keller and Rutkowski models strengthens the conceptual basis of the Keller/eGFR ratio, as they similarly capture a predictable, non-pathological reduction in kidney function with ageing [19].

**Fig. 1:**
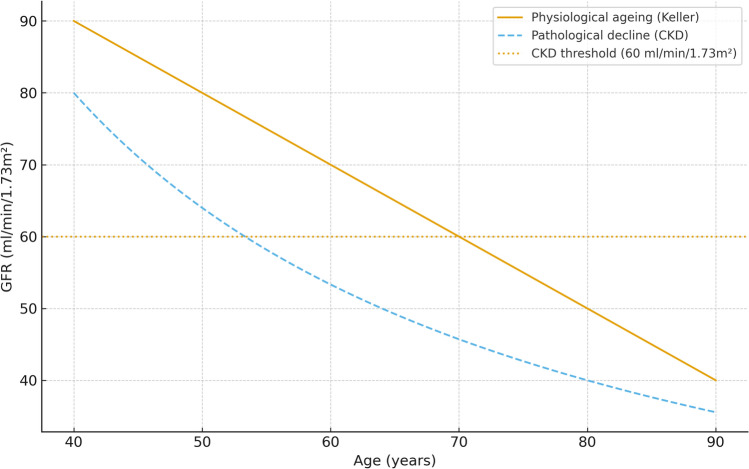
GFR trend: physiological vs CKD

**Fig. 2:**
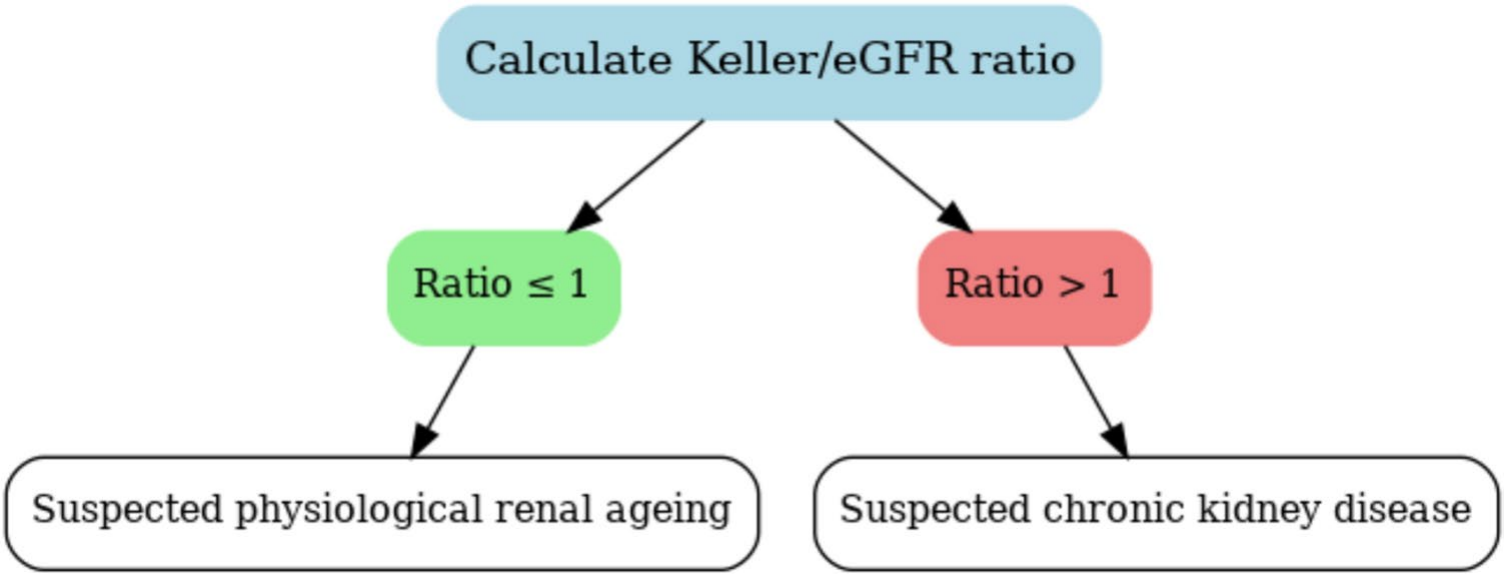
Decision algorithm based on the Keller/eGFR ratio. A ratio ≤ 1 suggests normal renal ageing, whereas a ratio > 1 indicates a reduction in GFR greater than expected for age, suggestive of underlying CKD

The concept has been further refined through the Keller/eGFR ratio, which compares the age-predicted GFR derived from Keller’s formula (the eGFR an old individual should have according to his/her age) with that estimated by standardized equations such as CKD-EPI (the eGFR an old individual has). This ratio allows two scenarios to be discriminated (Figs. 1, 2): a Keller/eGFR ratio ≤ 1, consistent with a probable normal renal aging without evidence of kidney disease; and a Keller/eGFR ratio > 1, indicating a greater reduction in GFR than expected for age and thus suggesting the presence of underlying CKD [20]. The Keller/eGFR ratio would be expected to approximate 1 in individuals undergoing physiological renal aging, as the Keller formula represents an idealized age-predicted GFR. In real-world conditions, however, Keller tends to modestly overestimate GFR relative to creatinine-based estimating equations such as CKD-EPI, which are affected by age-related sarcopenia and reduced creatinine generation. Consequently, ratios slightly below 1 are fully compatible with normal renal aging and fall within the expected range of analytical and biological variability. What is clinically meaningful is not the exact attainment of a ratio equal to 1, but rather a substantial deviation from the age-predicted estimate, which may suggest a pathological decline in GFR exceeding that attributable to aging alone. Accordingly, the Keller/eGFR ratio should be interpreted as a relative indicator of the discrepancy between expected and observed kidney function, rather than a strict diagnostic cut-off. It should be noted that the Keller formula provides a non–body surface area–indexed value (ml/min), whereas CKD-EPI is expressed in ml/min/1.73 m^2^. Although this difference may introduce a small proportional bias, the ratio maintains interpretive validity because it reflects the relative divergence between expected and observed GFR [21]. The clinical value of this index lies in its potential as a simple screening tool to reduce CKD overdiagnosis in older adults, a frequent occurrence when relying on CKD diagnosis solely on eGFR value [22]. The Keller/eGFR ratio may therefore be applied in population screening and large epidemiological databases. For further normal renal aging diagnosis, this index should be combined with distinctive markers of kidney damage such as increased albuminuria, imaging abnormalities, mineral disturbances, anemia, etc. to improve diagnostic accuracy [23]. Wider adoption of this tool could enable more accurate stratification of kidney function in the geriatric population, thereby reducing unnecessary medicalization and optimizing healthcare resource allocation [24].

Figure. 1 does not suggest that an eGFR of 30 ml/min in a 90-year-old is clinically benign; it illustrates the expected physiological trajectory of GFR decline at advanced age. Evidence indicates that in older adults the prognostic value of eGFR depends primarily on the presence of kidney damage markers rather than on the absolute eGFR level. Studies using measured GFR have shown that many individuals ≥75 years with eGFR 30–44 ml/min/1.73 m² but without albuminuria, anemia, mineral abnormalities or structural changes have stable kidney function and outcomes similar to peers with higher eGFR. Thus, low eGFR in isolation should not be equated with pathological decline, reinforcing the need for an integrative assessment rather than reliance on eGFR alone. 

Both groups may present with a reduced GFR. However, in normal renal ageing this decline is predictable and proportional to age (up to 1 ml/year), whereas in CKD the reduction is more pronounced (> 1 ml/min) and cannot be explained by ageing alone. Biochemical markers (Table 1) offer further discriminatory value in distinguishing normal renal ageing from CKD. In the context of ageing, serum creatinine and urea levels generally remain within normal limits, whereas in CKD these parameters are often elevated. Hemoglobin concentrations are usually preserved in individuals with renal ageing, but decline in CKD due to the onset of renal anemia. Similarly, abnormalities of mineral metabolism are absent in normal renal ageing but represent a hallmark of CKD, with hypocalcemia, hyperphosphatemia, and secondary hyperparathyroidism contributing to the risk of renal osteodystrophy and cardiovascular complications. Urinalysis and renal imaging provide additional diagnostic insights: while both tend to be unremarkable in normal renal ageing alone, CKD is frequently associated with proteinuria, microscopic hematuria, or structural changes such as nephroangiosclerosis, cortical thinning, parenchymal heterogeneity, renal asymmetry, and atrophy. Taken together, these features underscore that GFR in isolation is insufficient to discriminate renal ageing from CKD. A more robust diagnostic strategy requires integration of the Keller/eGFR ratio with biochemical indices, markers of kidney damage, and imaging findings, thereby reducing the risk of overdiagnosis and inappropriate treatment in old individuals [25, 26]. Recent evidence from Astley ME et al. [28] further reinforces the need to differentiate physiological age-related GFR decline from true chronic kidney disease (CKD) in older adults. In a large geriatric cohort, the authors showed that a substantial proportion of individuals with an eGFR of 45–59 ml/min/1.73 m^2^ exhibited no markers of kidney damage (absence of albuminuria, normal imaging findings, and preserved biochemical parameters), and had mortality and cardiovascular risk comparable to peers with eGFR ≥ 60. These findings support the concept of the “senescent kidney” as a phenotype distinct from CKD, closely aligned with our observations in subjects presenting a Keller/eGFR ratio ≤ 1. Astley also demonstrated that the rate of GFR decline in healthy older adults (~ 0.8–1 ml/min/year) closely matches the physiological trajectory predicted by the Keller formula, providing an important external validation for its use as an age-adjusted reference for expected GFR. Overall, the study by Astley strengthens our proposal that eGFR alone should not serve as the primary diagnostic criterion for CKD in the elderly. It underscores the importance of integrating markers of kidney damage (albuminuria, imaging abnormalities, mineral metabolism disturbances) to distinguish physiological renal ageing from true nephropathy. In this context, the Keller/eGFR ratio, when interpreted alongside these indicators, appears consistent with current international evidence and may help mitigate overdiagnosis and unnecessary medicalization in geriatric populations. Data from the Abruzzo cohort showed that combining the Keller/eGFR ratio with clinical and laboratory markers allows a clearer distinction between renal ageing and CKD, supporting its value on a broader scale [27]. An integrated diagnostic approach offers a more personalized assessment of CKD in old individuals, helping to reduce overdiagnosis and optimize both clinical management and healthcare resources.

**Table 1:** Differences between “Normal renal ageing and Chronic Kidney Disease—Stage 3”

	Normal renal ageing	Chronic kidney disease stage 3
Glomerular Filtration Rate	Normal or reduced	Reduced
Keller/eGFR	≤ 1	> 1
Creatininemia	Normal	High
Urea	Normal	High
Hemoglobin	Normal	Normal or reduced
Calcium-Phosphorus Metabolism	Normal	Altered
Urinalysis	Normal	Normal or altered
Renal Imaging	Normal with age-related cortical thinning	Altered

## Advanced GFR estimating equations in old people

New equations for GFR estimation have been developed to improve diagnostic accuracy in older adults, addressing the limitations of traditional formulas such as Cockcroft–Gault, MDRD, and CKD-EPI, which were derived from predominantly younger or mixed-age populations. The BIS-1 (Berlin Initiative Study) equation, published in 2012, was specifically designed for individuals aged ≥ 70 years. It is based on serum creatinine, age, and sex, and was derived from a large prospective German cohort [28]. BIS-1, calibrated specifically for the geriatric population, has demonstrated greater accuracy and reduced bias compared with CKD-EPI, particularly in the critical range of 30–60 ml/min/1.73 m^2^ [29]. However, its validation was conducted almost exclusively in European cohorts, raising concerns about its generalizability to more ethnically diverse populations. To address these limitations, the BIS-2 equation was developed, incorporating cystatin C alongside creatinine, age, and sex. The inclusion of cystatin C, which is less affected by muscle mass and dietary protein intake, improves the accuracy of GFR estimation and reduces diagnostic errors related to sarcopenia [30]. BIS-2 provides more accurate estimates than BIS-1 and CKD-EPI, particularly in frail older adults. Nonetheless, its use is limited by the influence of non-renal factors such as inflammation, obesity, and thyroid dysfunction, as well as by the cost and availability of cystatin C testing [13]. The EKFC equation (European Kidney Function Consortium equation) [9] was developed to provide a model applicable across the entire lifespan, from childhood to old age. It has been validated in a large European population against direct GFR measurements, including iohexol and iothalamate clearance [31].The EKFC equation does not require separate formulas for different age groups and reduces bias and variability, particularly in older individuals, where CKD-EPI and MDRD often misclassify aging decline as pathological. However, like other GFR estimating equations (MDRD, CKD-EPI, BIS, EKFC), it provides only a single numerical eGFR value without indicating its individual reliability, a limitation given that accuracy is affected by clinical factors (advanced age, sarcopenia, inflammation, comorbidities) and by the characteristics of the derivation cohorts. In this context, Lanot et al. [4] proposed an innovative approach using machine learning models to compare EKFC estimates with gold-standard GFR measurements (iohexol and inulin clearance), not only predicting the estimated value but also quantifying the probability that such an estimate was accurate. From this comparison, the authors derived a “confidence index,” reflecting the likelihood that the EKFC-estimated GFR corresponds to the true measured value in an individual patient. In practice, the equation provides not only a numerical estimate (e.g., eGFR = 48 ml/min/1.73 m^2^) but also an indicator of reliability, with a high index suggesting close agreement with the measured GFR and a low index signaling greater uncertainty. This approach offers two major advantages: it supports clinical personalization, guiding physicians to interpret low-confidence estimates with caution (e.g., by confirming with cystatin C or direct methods), and it enhances risk stratification in epidemiological databases by weighting estimates according to their reliability. The integration of such a confidence index into laboratory reporting—analogous to quality indicators in genetic or biochemical tests—could become a standard component of renal function assessment. Adoption of EKFC, enriched by artificial intelligence tools and validated in international multicenter cohorts, may thus represent a new global benchmark for GFR evaluation, particularly in the geriatric population, although further validation is needed in non-European cohorts and in complex clinical subgroups such as oncology, obesity, and frailty [4].

## Limitations and research perspectives

The assessment of kidney function in older adults remains a complex and debated issue. Although the currently available equations represent a major advance compared with earlier approaches, they are not without important methodological limitations. A key concern is the considerable variability across formulas: the same patient may be classified into a different CKD stage depending on whether MDRD, CKD-EPI, BIS, or EKFC is applied. This discrepancy reflects not only the distinct variables incorporated into the models (creatinine, age, sex, body weight, cystatin C), but also the characteristics of the populations in which these equations were originally derived and validated [32]. A limitation relates to the comorbidities common in older adults. Conditions such as diabetes mellitus, hypertension, heart failure, and metabolic syndrome influence true kidney function. At the same time, they affect the biomarkers used for its estimation, further complicating the accuracy of GFR equations in older people [16]. Systemic conditions such as chronic inflammation, obesity, sarcopenia, and thyroid dysfunction may also alter creatinine and cystatin C levels, thereby reducing the accuracy of GFR equations. This helps explain why, in frail older adults, the sole use of a creatinine-based equation often leads to CKD overdiagnosis, mislabeling individuals with simple renal ageing as renal diseased [28]. There is a clear need for prospective validation in multicenter cohorts. Most equations, including BIS-1, were developed and tested almost exclusively in European populations, with limited representation of other ethnic groups. Longitudinal studies are therefore essential to confirm their validity in more diverse populations and in complex clinical subgroups such as oncology patients, individuals with obesity, and frail older adults. At the same time, research is moving toward the integration of novel biomarkers for assessing kidney function and injury. Molecules such as NGAL (neutrophil gelatinase–associated lipocalin), KIM-1 (kidney injury molecule-1), and L-FABP (liver-type fatty acid binding protein) show promise in detecting early tubular damage and may complement traditional equations, enabling a more refined risk stratification [21]. Markers of cellular ageing (p16^INK4a, SA-β-gal, telomere length) are emerging as promising tools to differentiate normal from pathological renal functional decline [33]. An important challenge in older individuals is the prevention of overtreatment. Strict application of KDIGO criteria combined with reliance on all GFR estimating equations may lead to low-protein diets or unnecessary pharmacological interventions, with risks of sarcopenia, hyperkalemia, and impaired quality of life. Integrating the Keller/eGFR ratio with clinical, laboratory, and imaging markers can help limit these consequences, avoiding the misclassification of normal ageing as CKD. Traditional formulas such as Cockcroft–Gault are simple and widely used for drug dosing but are strongly influenced by body weight and inaccurate in sarcopenic patients. MDRD improved precision in CKD but tends to underestimate GFR in individuals with normal or mildly reduced function and in those over 70 years. CKD-EPI is now the reference standard and performs well at higher GFR values, but remains affected by age-related loss of muscle mass. BIS-1, developed for individuals ≥ 70 years, provides greater accuracy in older adults, although validated mostly in European cohorts. BIS-2, which incorporates cystatin C, further improves accuracy but is limited by cost and test availability. More recent formulas such as FAS and EKFC were designed for use across the full lifespan, while cystatin C–based equations (CKD-EPI CysC and CKD-EPI creatinine + cystatin C) show higher robustness in frail patients, albeit influenced by non-renal factors such as inflammation, obesity, and thyroid dysfunction. Direct GFR measurement methods (inulin, iohexol, iothalamate clearance) remain the gold standard for research purposes, but their technical complexity and high cost restrict routine clinical application [34]. Systematic comparisons show that no GFR equation is free of limitations. The choice of the most appropriate method must consider patient age, comorbidities, clinical context, and resource availability. Future perspectives should move toward an integrated, multimodal approach combining validated equations, novel biomarkers, imaging, and comprehensive clinical assessment, with the aim of reducing overdiagnosis, individualizing therapy, and optimizing CKD management in older individuals (Table 2).

**Table 2:** Comparison of GFR estimation methods in older adults

Equation/method	Main variables	Strengths	Limitations in the elderly
Cockcroft–Gault (1976)	Creatinine, age, sex, weight	Simple, historically used for drug dosing	obesity; -frailty/sarcopenia
MDRD (1999)	Creatinine, age, sex, race	Better than C–G in CKD	Underestimates GFR in normal/mild impairment; less accurate > 70y
CKD-EPI (2009/2021)	Creatinine, age, sex (race only in 2009 version)	Current guideline standard; better at higher GFR	Still influenced by muscle mass loss in elderly
BIS-1 (2012)	Creatinine, age, sex	Specifically developed for ≥ 70 years	Limited validation outside European cohorts
BIS-2 (2012)	Creatinine, cystatin C, age, sex	Higher accuracy in elderly; less muscle-dependent	Cystatin C assays not always available; cost
FAS (2016)	Creatinine, age, Q-value (median creatinine of healthy subjects)	Valid across full age spectrum (children–elderly)	Needs further validation in very old/frail patients
EKFC (2021)	Creatinine, age, sex	Pan-European validation; robust across ages	Still limited experience in clinical practice
CKD-EPI Cystatin C (2012)	Cystatin C, age, sex	Less affected by sarcopenia	Influenced by inflammation, thyroid disorders, obesity
CKD-EPI Creatinine + Cystatin C	Creatinine, cystatin C, age, sex	Most accurate in mixed populations	Higher cost; limited availability in some labs
Measured clearance (inulin, iohexol, iothalamate)	Direct measurement	Gold standard; research reference	Expensive, time-consuming; not practical in routine use
24 h Creatinine clearance	Serum and urine creatinine	Widely available, simple	Inaccurate due to collection errors; muscle mass dependent

## Conclusion

Distinguishing normal renal ageing from chronic kidney disease (CKD) in older adults is an increasingly important clinical and epidemiological challenge. The progressive decline in glomerular filtration rate (GFR), averaging about 1 ml/min/1.73 m^2^ per year from the fourth decade of life, should not automatically be interpreted as CKD. In most individuals, this reduction reflects just renal ageing, unaccompanied by biochemical, urinary, or imaging abnormalities. However, commonly used solely eGFR values based on creatinine-based equations (MDRD, CKD-EPI) for CKD diagnosis in older people, usually lead to CKD overdiagnosis and consequently inappropriate interventions such as low-protein diets or antifiltration therapies (ACE inhibitors, ARBs) with potential adverse effects including accelerated sarcopenia, electrolyte disorders, and impaired quality of life. In this setting, the Keller/eGFR ratio represents a simple, easily applicable tool to help differentiate age-related from pathological GFR decline: values ≤ 1 are consistent with possible normal renal ageing, while values > 1 suggest underlying CKD. Nonetheless, this ratio should not be used in isolation but rather as a complement to clinical parameters, laboratory markers (proteinuria, mineral metabolism, anemia), and renal imaging—it allows a more accurate assessment of kidney function in older adults and reduces the risk of inappropriate medicalization. The present analysis was not based on individual-level clinical data and therefore cannot provide diagnostic accuracy measures such as sensitivity, specificity or ROC curves. The Keller/eGFR ratio should consequently be viewed as a conceptual and exploratory tool whose empirical performance needs to be evaluated in future studies including albuminuria, imaging and biochemical markers of kidney damage.

## Data Availability

No datasets were generated or analyzed during the current study.
